# The Cost-Effectiveness of Lenvatinib in the Treatment of Advanced or Unresectable Hepatocellular Carcinoma from a Canadian Perspective

**DOI:** 10.1155/2021/8811018

**Published:** 2021-02-23

**Authors:** Brandon M. Meyers, Arndt Vogel, Paul Marotta, Petr Kavan, Laveena Kamboj, Janice Pan, Marc Geadah, David Trueman, Suthakar Sabapathy

**Affiliations:** ^1^Department of Oncology, Juravinski Hospital and Cancer Centre, Hamilton Health Sciences, McMaster University, Hamilton, Canada; ^2^Department of Gastroenterology, Hepatology and Endocrinology, Medical School Hannover, Hannover, Germany; ^3^Multi-Organ Transplant Program, London Health Sciences Center, The University of Western Ontario, London, Ontario, Canada; ^4^Department of Oncology, Faculty of Medicine, McGill University, Montreal, Quebec, Canada; ^5^Eisai Ltd, Mississauga, Ontario, Canada; ^6^Eisai Inc., Woodcliff Lake, NJ, USA; ^7^PIVINA Consulting Inc, Mississauga, Ontario, Canada; ^8^Source Health Economics, London, UK

## Abstract

Lenvatinib is an oral multikinase inhibitor indicated for the first-line treatment of unresectable hepatocellular carcinoma (uHCC). In the Phase III REFLECT trial, lenvatinib was noninferior in the primary endpoint of overall survival versus sorafenib, the only systemic therapy funded in Canada prior to the introduction of lenvatinib. Lenvatinib also demonstrated statistically significant improvement compared to sorafenib in secondary endpoint progression-free survival, time to progression, and objective response rate. The aim of this analysis was to estimate the cost-effectiveness of lenvatinib versus sorafenib for the first-line treatment of patients with uHCC from a Canadian perspective. A cost-utility analysis was conducted using partitioned survival modelling, with health states representing progression-free disease, progressed disease, and death. Health effects were measured using quality-adjusted life years (QALYs), and costs were represented in Canadian dollars. Clinical inputs were derived from the REFLECT trial, with outcomes extrapolated using parametric survival models. EQ-5D data collected in REFLECT were used to determine health state utility values, and estimates of resource use came from a survey of clinicians. The model predicted incremental costs of-$5,021 and incremental QALYs of 0.17, making lenvatinib dominant over sorafenib. The model demonstrates lenvatinib to be a cost-effective use of resources versus sorafenib in Canada for the treatment of uHCC. Overall costs are lower compared with sorafenib, while health benefits are greater, with modelled progression-free and overall survival extended by 4.1 and 2.6 months in the lenvatinib arm, respectively.

## 1. Introduction

Hepatocellular carcinoma (HCC) accounts for approximately 90% of liver cancers globally [1] and approximately 72% in Canada [2]. It is estimated that 3,000 Canadians were diagnosed with liver cancer in 2019, with around 1,400 deaths [3], reflecting the poor survival rate, approximately 20% over five years [2].

Prognosis is dependent on liver function, performance status, and tumor type [4]. The goal of tumor staging in HCC is to estimate a patient's prognosis, allowing for an appropriate therapy to be administered [5]. There is no universally adopted staging system for HCC [4]; however, the Barcelona Clinic Liver Cancer (BCLC) staging system is commonly used [6]. Early stage disease (BCLC stage 0 and A) is managed with curative therapies, whereas in advanced disease (BCLC stage B or C, in patients ineligible for locoregional therapies), the mainstay is systemic therapy.

Prior to the introduction of lenvatinib, sorafenib was the only systemic therapy publicly funded for the first-line treatment of HCC in Canada, and regorafenib is the only publicly funded second-line treatment. Lenvatinib, an oral multikinase inhibitor, was recommended for funding by the pan-Canadian Oncology Drug Review (pCODR) in July 2019 [7] for the first-line treatment of adult patients with unresectable HCC (uHCC). This recommendation is based on results from the REFLECT trial [8], the first positive trial in over 10 years in the first-line HCC treatment landscape [9–16].

The REFLECT trial was an open-label, phase III, multicentre, noninferiority trial that evaluated the efficacy and safety of lenvatinib versus sorafenib in patients with uHCC. Lenvatinib showed noninferiority versus sorafenib, with median overall survival (OS) duration for lenvatinib of 13.6 months (95% confidence interval: 12.1, 14.9) compared with 12.3 months for sorafenib (95% confidence interval: 10.4, 13.9; hazard ratio: 0.92) [8]. Lenvatinib demonstrated statistically significant improvement compared with sorafenib for all secondary efficacy endpoints including progression-free survival (PFS; 7.4 vs. 3.7 months), time to progression (8.9 vs. 3.7 months), and objective response rate by mRECIST (24% vs. 9%) [8]. A masked independent review was conducted of PFS and objective response rate in accordance with Food and Drug Administration recommendations. Results were consistent with the investigator review, validating the findings of the REFLECT trial [17].

Liver cirrhosis is identified in the majority of patients with HCC [18], influencing pharmacokinetics and increasing both side effects and hepatotoxicity [19, 20]. Additionally, adverse events (AEs) can influence tolerability; sorafenib has been associated with higher rates of palmar-plantar erythrodysesthesia, also known as hand-foot syndrome, a debilitating inflammation of the skin. Lower rates of hand-foot syndrome and diarrhea were observed in the lenvatinib arm of REFLECT [8].

The pCODR Expert Review Committee (pERC) in its recommendation highlighted the need for effective and more tolerable treatment options in first-line uHCC. The committee noted that “*toxicities observed with lenvatinib (i.e., hypertension) are more easily managed than those seen with sorafenib (i.e., hand-foot syndrome)*” [7]. The pCODR Clinical Guidance Panel report also highlighted that hypertension can be managed with antihypertensive medications and usually does not cause symptoms, whereas hand-foot syndrome can affect daily activities such as standing and walking [21]. pERC concluded that lenvatinib “*aligned with patient values of having a treatment option that offers different and potentially more manageable toxicities compared to sorafenib*” [7].

Cost-utility analyses guide policy making by estimating the cost-effectiveness associated with the introduction of new health technologies. An incremental cost-utility ratio is an expression of the ratio of benefits to costs and is calculated by dividing the incremental costs of a new technology by the incremental benefits.

Economic evaluations form a core part of health technology assessment in Canada, where cancer drugs are assessed through the Canadian Agency for Drugs and Technologies in Health's pCODR. The Economic Guidance Panel at pCODR which reviewed the cost-effectiveness of lenvatinib compared with sorafenib considered the submitted model structure appropriate and agreed with the majority of assumptions made in the base-case [21].

Since the pERC recommendation, the cost-utility analysis submitted for that review has been updated to better reflect the Canadian HCC environment. Specifically, two additional factors are explored in this manuscript:

  (1) Reducing the treatment cost of sorafenib to estimate the confidential listing agreements that may exist between the manufacturer and Canadian payers
  (2) Adjusting OS for the imbalance in postprogression therapies and assuming that only regorafenib (the only product publicly funded in second line) is used after lenvatinib or sorafenib

The aim of this study was to determine, from the Ministry of Health perspective, the cost-utility of lenvatinib versus sorafenib for the first-line treatment of adult patients with uHCC in Canada.

## 2. Materials and Methods

A pharmacoeconomic model was constructed using efficacy and safety data from the REFLECT trial [8]. Health effects were measured using quality-adjusted life years (QALYs), which consider both the quantity and quality of life generated by the new drug treatment, and cost-effectiveness was expressed as a ratio of cost per QALY gained. Consistent with REFLECT, the model included two doses of lenvatinib based on body weight: 8 mg (two 4 mg capsules) given orally once daily for patients with a body weight of <60 kg and 12 mg (three 4 mg capsules) given orally once daily for patients with a body weight of  ≥60 kg. The comparator was a regimen of 400 mg twice daily of sorafenib, the only other systemic therapy available for first-line treatment of uHCC in Canada.

Costs considered were those associated with treatment and healthcare resource utilization, and the impact of societal costs, including lost productivity hours, was considered in the scenario analysis. Outcomes were modelled over a 10-year time horizon with costs and QALYs discounted at a rate of 1.5%, as per the Canadian Agency for Drugs and Technologies in Health's Guidelines for the Economic Evaluation of Health Technologies [22]. A cycle length of 28 days was used, and half-cycle correction was implemented using the life table method.

Model assumptions and inputs were reviewed by clinical experts, and the model was updated to ensure alignment with Canadian clinical practice.

**Figure 1:**
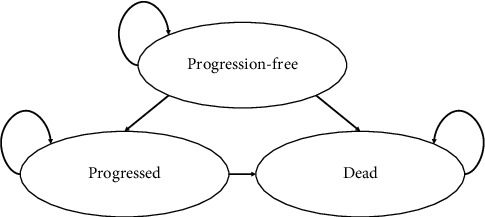
Model health states.

The analysis used a partitioned survival model comprised of a series of health states (in this case “progression-free,” “progressed,” and “dead, ”see Figure 1), each associated with different costs and utility values. The partitioned survival model approach is commonly used in evaluating interventions for advanced or metastatic cancers [23].

The proportion of patients in each health state over time was estimated using results from the REFLECT study [8], and extrapolation was performed to provide estimates at time points beyond the trial period. The OS curve (representing all patients that are alive) was partitioned into PFS and progressed disease states (representing patients without and with worsening/spreading of the cancer, respectively). In REFLECT, progression was defined based on investigators' tumor response evaluations according to mRECIST for HCC for hepatic lesions. Post hoc exploratory analyses using mRECIST and RECIST 1.1 based on an independent imaging review were also conducted and produced similar results [24].

The economic evaluation included adults with untreated advanced or unresectable HCC and Child-Pugh class A liver function. Population data and results from the full analysis set in the REFLECT trial were used. The full analysis set included all randomized patients and was the primary analysis set for all efficacy evaluations. Most patients had Barcelona Clinic Liver Cancer stage C disease (79%), although some were stage B (21%, those who were considered ineligible for transarterial chemoembolization). In total, 99% of patients in REFLECT had Child-Pugh class A liver function (the remainder were Child-Pugh class B) [8].

At the data cutoff of 13th November 2016, 73.4% of patients in the lenvatinib arm and 73.5% of patients in the sorafenib arm had died, necessitating extrapolation beyond the end of REFLECT for the OS and PFS endpoints. This extrapolation was achieved using parametric survival analysis, following guidance from the National Institute for Health and Care Excellence Decision Support Unit [23]. The proportions of subjects who remained on treatment over time (0% and 4% for lenvatinib and sorafenib, respectively) were taken from the Kaplan–Meier estimator.

Parametric survival models were used to extrapolate outcomes, and covariate analyses were performed to evaluate baseline factors that may have impacted OS in the overall study population, including alpha fetoprotein and HCC etiology. The covariates that were included in the analysis were originally chosen from clinician input and refined using a backwards stepwise selection procedure. The model was adjusted for baseline imbalances. Covariates included (but were not limited to) baseline alpha fetoprotein (concentration ≥ or < 200 ng/mL), body weight (<60, ≥60 kg), and Child-Pugh score.

The choice of survival distribution for extrapolation was based on statistical goodness of fit (using the Akaike information criterion and the Bayesian information criterion), clinical plausibility, and consistency with previous findings of extrapolation in advanced HCC. The log-logistic model used for the base-case OS in both arms had the best AIC and BIC and was similar in nature to the log-normal distribution used in previous appraisals. Log-normal was used for both arms for PFS. The gamma distribution was associated with the best AIC and BIC scores for the sorafenib arm but was ruled out due to clinically implausible extrapolations. The impact of using alternative survival was explored in scenario analysis (see Supporting Information).

A time horizon of 10 years was included in the base-case and was considered sufficient to capture all outcomes, with 1.7% and 1.1% of modelled patients remaining alive at 10 years in the lenvatinib and sorafenib arms, respectively.

Utility values represent the strength of individuals' preferences for different states of health and typically range between zero (representing death) and one (representing full health). When clinical trials collect utility data, this is usually in the form of a questionnaire completed by patients at different time points, with responses converted to utility values using appropriate tariffs [25].

In REFLECT, patients completed the commonly used EQ-5D-3 L questionnaire at the Baseline visit, on Day 1 of each subsequent treatment cycle, and at the Off-Treatment visit. The UK EQ-5D-3 L tariff was applied to responses at each time point to generate utility values [26].

Overall, EQ-5D-3 L data from REFLECT were analysed and compared with EQ-5D data based on the lenvatinib and sorafenib arms separately. A linear mixed model was also used to compare after controlling for patient characteristics. These adjusted mean utility values were similar between the arms, with a numerical difference in favor of lenvatinib in both the progression-free and progressed health states. Disutilities associated with AEs were not explicitly modelled; it was assumed that the utility values implicitly included AEs because a proportion of patients in each health state were experiencing AEs at any given time. It was therefore conservatively assumed that utility values for both arms were equal, and so the utility scores from the full REFLECT population were used (0.745 in the progression-free health state and 0.678 in the progressed health state). Analyses using utility values from other pCODR uHCC recommendations were also performed (see Supporting Information).

All costs were valued in 2019 Canadian Dollars, and unit costs were aggregated from multiple public sources [27–31]. The price included in the model for sorafenib was sourced from the Ontario Ministry of Health and Long-Term Care Exceptional Access Program Formulary [32], and the price for lenvatinib was supplied by the manufacturer. The price for regorafenib was sourced from pCODR's Economic Guidance Report for regorafenib [30]. Total drug costs were calculated based on the target dose per day observed in the REFLECT trial [8] adjusted by the dose intensity.

In the absence of evidence from either the REFLECT trial or the published literature, estimates of resource use in each of the health states and for management of AEs were informed by a resource use survey completed by six clinical experts.

Adverse events considered in the economic model included grade 3 or 4 treatment-emergent AEs occurring in ≥5% of patients in either treatment arm of the REFLECT trial. Additional grade 3 or 4 treatment-emergent AEs that occurred in <5% of patients in either treatment arm were included if they were identified as being significant either clinically or economically.

The cost of death was included at the time of death, based on the cost associated with the use of palliative services by patients in Ontario between 2002 and 2003 for gastrointestinal cancers (excluding colorectal cancer) [33].

Table 1 presents an overview of utility values and selected costs included in the model.

Two factors were considered in key scenario analyses to better reflect the Canadian HCC environment. These are described in further detail below.

### 2.1. Sorafenib Treatment Drug Price

In the pCODR pERC recommendation, the only condition for reimbursement was that “the public drug plan cost of treatment with lenvatinib should not exceed the public drug plan cost of treatment with sorafenib.”

In the base-case, the treatment drug price of sorafenib ($46.47 per 200 mg) was taken from the EAP Formulary. As jurisdictions listed sorafenib over 10 years ago, some jurisdictions may or may not have confidential agreements in place. Nonetheless, this was considered by assuming the net price of sorafenib would be reduced by 5%, 10%, and 15% in scenario analyses.

### 2.2. Postprogression Therapy

The postprogression therapy scenario combined two changes to the model to reflect the publicly funded second-line treatment in Canada and the imbalance in postprogression therapies in REFLECT.

Firstly, in the pCODR recommendation, pERC acknowledged that “*it is reasonable to use second-line regorafenib after lenvatinib*.” In the model base-case, only therapies publicly funded for this indication (sorafenib and regorafenib) were considered as postprogression therapies. However, in this scenario, all patients with progressed disease who received either sorafenib or regorafenib in REFLECT, 108 (31%) patients in the lenvatinib arm and 52 (15%) patients in the sorafenib arm, were assumed to receive regorafenib. Duration of treatment with regorafenib was based on the RESORCE trial (median of 3.6 months) [34].

Secondly, when adjusting for the use of postprogression therapies, the OS hazard ratio (95% confidence interval) for lenvatinib versus sorafenib was 0.87 (0.75, 1.01). This adjustment was included in this scenario to adjust for the extended OS that might be present in patients receiving postprogression therapies. The pCODR Economic Guidance Panel considered a similar analysis adjusting for this imbalance and considered it appropriate [21].

Joint parameter uncertainty was explored through probabilistic sensitivity analysis, in which all parameters were assigned distributions and varied jointly across 5,000 Monte Carlo simulations to produce an overall incremental cost-utility ratio.

Scenario analyses were performed in which key structural assumptions were varied. Key scenario analyses are presented in the Results section, and additional scenario analyses are presented in the Supporting Information.

Parameter uncertainty was tested using univariate sensitivity analysis, in which all model parameters were varied over a plausible range, and the net monetary benefit calculated [35]. The 10 most influential parameters on the net monetary benefit were displayed in a tornado diagram (see Supporting Information).

## 3. Results

In the base-case, lenvatinib was associated with incremental costs of –$5,021, incremental QALYs of 0.17, and was therefore considered dominant versus sorafenib. Results of key scenario analyses adopted to reflect the Canadian HCC landscape were consistent with this conclusion (Table 2). Assuming a conservative price reduction of 15% for sorafenib and the utilization of postprogression treatment of regorafenib, lenvatinib remained dominant compared to sorafenib.

**Figure 2:**
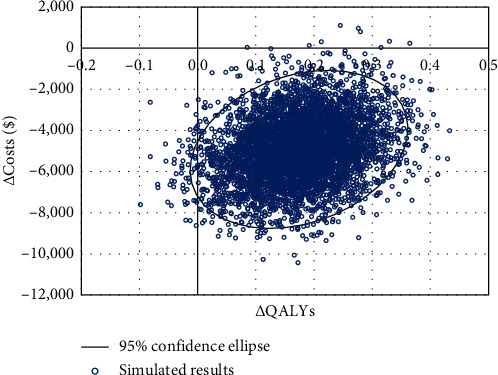
Cost-effectiveness plane. Abbreviations: QALYs, quality-adjusted life years.

The results of 5,000 simulations were plotted on the cost-effectiveness plane (Figure 2). The average incremental savings over the simulated results were $4,921, and the average incremental QALYs gained were 0.17; this is highly congruent with deterministic changes in costs and QALYs of –$5,021and 0.17, respectively. 100% of simulations were considered cost-effective at a threshold of $50,000 per QALY.

Descriptions and results of other scenario analyses undertaken, in addition to univariate sensitivity analysis results, are presented in the Supporting Information.

## 4. Conclusions

Assuming list prices for lenvatinib and sorafenib, lenvatinib is dominant, as it confers greater health benefits and incurs fewer costs, therefore making it a cost-effective use of resources in Canada. Lenvatinib remains dominant when a 15% sorafenib price reduction is applied. Of note was the fact that all scenarios align with the base-case by demonstrating lenvatinib to be dominant versus sorafenib (see Supporting Information) [21]. pCODR considered the submitted model to be appropriate, with the assumptions made and related input variables causing little variation in the results.

The reduction in overall costs observed in the model was driven by reduced primary drug costs in the lenvatinib arm. The incremental benefit favoring lenvatinib was likely driven by delayed progression, increasing time spent in the progression-free health state, which was associated with a higher utility value.

Lenvatinib may also be beneficial for patients who have not progressed on sorafenib but are intolerant, and pERC noted that it would be reasonable to consider switching these patients onto lenvatinib [7]. Furthermore, pCODR acknowledged that it would be reasonable to use second-line regorafenib following progression with lenvatinib. Lenvatinib remained dominant in a scenario incorporating this assumption (Scenario 5; Table 2), which was designed to conservatively reflect the Canadian HCC landscape. In addition to the inclusion of regorafenib only as a second-line treatment option, this scenario included a 15% rebate on the sorafenib price and adjustment of OS for the imbalance in postprogression therapies.

Although there are no data on the sequence of lenvatinib followed by regorafenib, pCODR made this recommendation based on a significant unmet need for second-line options following first-line systemic therapy and the similarity of the mechanisms of action of lenvatinib and sorafenib [7]. This is consistent with other publications, including those made by the Canadian Gastrointestinal Oncology Evidence Network (CGOEN), representing HCC experts nationally and the most recent ASCO JCO guidelines on HCC [36–39].

Another factor to consider when choosing between lenvatinib and sorafenib is the occurrence of AEs, some of which may have prognostic significance. As discussed, sorafenib has been associated with higher rates of hand-foot syndrome and diarrhea, with lower rates observed in the lenvatinib arm of REFLECT [8]. Several small and mostly retrospective analyses have identified a positive correlation between these AEs and TTP or OS in patients treated with sorafenib [40], and a post hoc analysis of REFLECT found that hypertension, diarrhea, proteinuria, and hypothyroidism were significantly associated with longer OS in patients treated with lenvatinib [41]. Therefore, the occurrence of these AEs may serve as useful indicators for predicting treatment benefit.

One notable feature of REFLECT was an imbalance in postprogression therapies between arms; more patients in the sorafenib arm received postprogression therapies, as lenvatinib delayed progression, meaning that at any given time fewer patients in the lenvatinib arm required postprogression therapy. In addition, patients on lenvatinib were often ineligible to enrol in second-line trials. Although fewer patients in the lenvatinib arm received postprogression therapies, more patients in the sorafenib arm received off-label drugs and/or therapies being evaluated in clinical trials. Nevertheless, the adjustment for postprogression therapies (which was designed to balance the impact of postprogression therapies on OS between arms) resulted in an increase in the net clinical benefit of lenvatinib. Incremental QALYs increased from 0.17 in the base-case to 0.22, and incremental survival rose from 2.6 to 3.6 months.

Imbalances in baseline characteristics may have resulted in underestimation of the OS benefit of lenvatinib. Indeed, lenvatinib was nominally superior to sorafenib after adjusting for baseline alpha fetoprotein (hazard ratio: 0.856; 95% confidence interval: 0.736, 0.995; *p*=0.0342) [42].

A limitation of the analysis is that it may have failed to adequately capture the reduced health-related quality of life experienced postprogression and the full benefit associated with lenvatinib, due to the small difference between pre- and postprogression utility values in REFLECT. The EQ-5D collection schedule meant that the postprogression measurement was taken shortly after progression, so it did not capture declining health-related quality of life thereafter.

In REFLECT, 1% of patients in each arm had Child-Pugh class B liver function, which is associated with a substantially poorer prognosis compared with Child-Pugh class A [43]. Data on the use of sorafenib in patients with Child-Pugh class B liver function are limited to observational studies that do not allow firm conclusions to be made; as expected, they show shorter OS for Child-Pugh B patients versus those with Child-Pugh A [44]. However, neither sorafenib nor lenvatinib is reimbursed in Canada for Child-Pugh B patients.

Multivariable parametric models were used to generate predictions for outcomes within the model; however, robust data are not available on the prognostic effect of some baseline variables. To account for these evidence gaps, a list of candidate baseline characteristics was presented to practicing medical oncologists with expertise in the management of HCC. They were asked to identify variables which they considered prognostic of outcomes in patients with uHCC who had not previously received systemic treatment.

The analysis presented here demonstrates that, in addition to providing a net clinical benefit, lenvatinib is expected to be cost saving in Canada for the treatment of advanced HCC. The model was robust to testing of assumptions with all scenario analyses aligning with the base-case conclusion of dominance versus sorafenib.

The positive funding recommendation of lenvatinib by pCODR in Canada provides patients with a new treatment option with potentially more manageable side effects, which has been demonstrated to be noninferior to sorafenib in terms of OS, with statistically significant improvements in PFS, time to progression, and objective response rate.

## Figures and Tables

**Table 1:** Utility values and selected costs.

Input	Value
Health state utility values	
Progression-free	0.745
Progressed	0.678

Drug therapy costs (per cycle)	
Lenvatinib	$2,142
Sorafenib	$4,320

Medical resource use costs (per cycle)	
Physician visits (progression-free)	$176
Physician visits (progressed)	$187
Laboratory tests (progression-free)	$29
Laboratory tests (progressed)	$23
Radiological tests (progression-free)	$131
Radiological tests (progressed)	$79
Hospitalisation (progression-free)	$27
Hospitalisation (progressed)	$80

Adverse event management costs (per event)	
Aspartate aminotransferase increased	$443
Asthenia	$1,879
Blood bilirubin increased	$1,954
Diarrhea	$384
Fatigue	$452
Gamma-glutamyl transferase increased	$418
Hypertension	$482
Palmar-plantar erythrodysesthesia	$418
Platelet count decreased	$418
Proteinuria	$418
Weight decreased	$1,845

Other costs	
Mortality cost (applied at time of death)	$31,583

**Table 2:** Base-case and key scenario results.

	Incremental costs	Incremental QALYs	Incremental LYs	Incremental progression-free years	ICUR
Base-case	–$5,021	0.17	0.22	0.34	Dominant
Key scenarios					
(1) 5% sorafenib price reduction	–$3,770	0.17	0.22	0.34	Dominant
(2) 10% sorafenib price reduction	–$2,518	0.17	0.22	0.34	Dominant
(3) 15% sorafenib price reduction	–$1,267	0.17	0.22	0.34	Dominant
(4) Adjustment for postprogression therapies and regorafenib only postprogression	–$9,472	0.22	0.29	0.34	Dominant
(5) Scenarios 3 and 4 combined	–$5,222	0.22	0.29	0.34	Dominant

Abbreviations: ICUR, incremental cost-utility ratio; Lys, life years; QALYs, quality-adjusted life years.

## Data Availability

The data used to support the findings of this study are included within the article and Supplementary Information file.

## Supplementary Materials

Supplementary Materials Disaggregated model outcomes and costs, full-scenario analysis results, and univariate sensitivity analysis results are provided as supplementary materials.
