# Supplementary material for: The Cost-Effectiveness of Lenvatinib in the Treatment of Advanced or Unresectable Hepatocellular Carcinoma from a Canadian Perspective
Source: Can J Gastroenterol Hepatol. 2021 Feb 23;2021:8811018. doi: 10.1155/2021/8811018 (PMC7929650; doi:10.1155/2021/8811018)
Supplement: Supplementary Materials — Disaggregated model outcomes and costs, full-scenario analysis results, and univariate sensitivity analysis results are provided as supplementary materials. [file 8811018.f1.docx]

Supporting Information

Appendix 1: Disaggregated model outcomes and costs

Table S1: Summary of outcomes

|  | **LEN** | **SOR** |
| --- | --- | --- |
| Progression-free years | 0.89 | 0.55 |
| LYs | 1.69 | 1.47 |
| QALYs | 1.20 | 1.03 |

Abbreviations: LEN, lenvatinib; LY, life year; QALY, quality-adjusted life year; SOR, sorafenib.

Table S2: Summary of costs

| **Cost category** | **LEN** | **SOR** |
| --- | --- | --- |
| Primary drug costs | $20,022 | $28,337 |
| Post-progression drug costs | $5,204 | $2,861 |
| Medical resource use costs | $8,056 | $7,024 |
| Adverse event costs | $614 | $440 |
| Mortality costs | $30,338 | $30,593 |
| **Total costs** | **$64,234** | **$69,255** |

Abbreviations: LEN, lenvatinib; SOR, sorafenib.

Appendix 2: Full scenario analysis results

Societal perspective

The model base-case was conducted using a Canadian Ministry of Health (public payer) perspective as per CADTH guidelines. In the societal perspective scenario, for each non-hospitalization health care resource used (within each health state and per AE event), one half-day of work hours was assumed to be lost. For each hospitalization, the average duration of stay, as per the Canadian Institute for Health Information Patient Cost Estimator [33] was considered in the calculation for work hours lost, which were multiplied by the average hourly salary in Canada in 2018 ($28.21). This scenario reduces the difference in costs between treatments but has no impact on incremental QALYs. Lenvatinib remains dominant over sorafenib (Table 3).

Table S3: Societal perspective scenario

| **Drug** | **Total Costs** | **Total QALYs** | **∆ Costs** | **∆ QALYs** | **ICUR** |
| --- | --- | --- | --- | --- | --- |
| Lenvatinib | $70,718 | 1.20 | -$4,161 | 0.17 | Dominant |
| Sorafenib | $74,879 | 1.03 | - | - | - |

Equal resource use between health states (progressed = progression-free)

In this scenario, medical resource use is the same regardless of whether patients are in the progression-free or progressed health states. This was implemented following clinical feedback suggesting that medical resource use may not vary. This scenario has almost no impact on incremental costs (the difference is reduced by $11), and incremental QALYs remain the same as the base-case. Lenvatinib remains dominant over sorafenib (Table 4).

Table S4: Resource use scenario

| **Drug** | **Total Costs** | **Total QALYs** | **∆ Costs** | **∆ QALYs** | **ICUR** |
| --- | --- | --- | --- | --- | --- |
| Lenvatinib | $64,169 | 1.20 | -$5,010 | 0.17 | Dominant |
| Sorafenib | $69,180 | 1.03 | - | - | - |

5-year and 3-year time horizon

The model base-case has a time horizon of 10 years. Alternative time horizons of 5 years and 3 years are considered below. Both scenarios increase the difference in costs between treatments and reduce the difference in QALYs. Lenvatinib remains dominant over sorafenib in both cases (Table 5).

Table S5: Time horizon scenarios

| **Drug** | **Total Costs** | **Total QALYs** | **∆ Costs** | **∆ QALYs** | **ICUR** |
| --- | --- | --- | --- | --- | --- |
| **5-year time horizon** | | | | | |
| Lenvatinib | $62,302 | 1.11 | -$5,608 | 0.14 | Dominant |
| Sorafenib | $67,910 | 0.97 | - | - | - |
| **3-year time horizon** | | | | | |
| Lenvatinib | $58,864 | 0.99 | -$6,385 | 0.11 | Dominant |
| Sorafenib | $65,248 | 0.88 | - | - | - |

0% and 3% discount rates

A 1.5% discount rate for costs and health outcomes is used in the base-case, as per CADTH guidelines. These scenarios explore the impact of lowering and raising the discount rate. Removing discounting decreases the difference in costs between arms but increases incremental QALYs. Increasing the discount rate to 3% increases the difference in costs between arms but has a minimal impact on incremental QALYs. Lenvatinib remains dominant over sorafenib in both cases (Table 6).

Table S6: Discounting scenarios

| **Drug** | **Total Costs** | **Total QALYs** | **∆ Costs** | **∆ QALYs** | **ICUR** |
| --- | --- | --- | --- | --- | --- |
| **0.0% discounting** | | | | | |
| Lenvatinib | $65,446 | 1.24 | -$4.871 | 0.18 | Dominant |
| Sorafenib | $70,318 | 1.06 | - | - | - |
| **3.0% discounting** | | | | | |
| Lenvatinib | $63,088 | 1.17 | -$5.158 | 0.17 | Dominant |
| Sorafenib | $68,246 | 1.01 | - | - | - |

Parametric survival analysis adjustments

The model base-case was based on multivariable adjustments to the PFS and OS curves. One scenario analysis is presented based on unadjusted parametric models (based on an intention-to-treat [ITT] approach). Another scenario analysis is presented that adjusts for AFP only (in addition to stratification factors), which is intended to provide an intermediary scenario to the multivariable (base-case) and unadjusted (scenario) analysis. Both scenarios result in increased incremental costs between treatments. When no adjustment is made, incremental QALYs decrease. When adjustment is made for AFP only, there is minimal impact on incremental QALYs. Lenvatinib remains dominant over sorafenib in both cases (Table 7).

Table S7: Baseline imbalance adjustment scenarios

| **Drug** | **Total Costs** | **Total QALYs** | **∆ Costs** | **∆ QALYs** | **ICUR** |
| --- | --- | --- | --- | --- | --- |
| **No adjustments to parametric survival analysis** | | | | | |
| Lenvatinib | $64,454 | 1.28 | -$6,849 | 0.11 | Dominant |
| Sorafenib | $71,302 | 1.17 | - | - | - |
| **Adjustment for AFP only in parametric survival analysis** | | | | | |
| Lenvatinib | $64,364 | 1.24 | -$5,685 | 0.17 | Dominant |
| Sorafenib | $70,049 | 1.07 | - | - | - |

Survival extrapolation scenarios

In the model base-case, the log-logistic distribution was used for both lenvatinib and sorafenib survival curves. These scenarios explore alternative distributions for extrapolation of OS and PFS. Incremental costs are greatest when the gamma distribution is used for PFS and are smallest when the log-logistic distribution is used. In the latter scenario, incremental QALYs are higher than in the base-case. Incremental QALYs are lowest when the gamma distribution is used for OS. In all case lenvatinib remains dominant over sorafenib (Table 8).

Table S8: Survival extrapolation scenarios

| **Drug** | **Total Costs** | **Total QALYs** | **∆ Costs** | **∆ QALYs** | **ICUR** |
| --- | --- | --- | --- | --- | --- |
| **Log-normal parametric distribution for OS** | | | | | |
| Lenvatinib | $64,359 | 1.20 | -$5,000 | 0.17 | Dominant |
| Sorafenib | $69,360 | 1.02 | - | - | - |
| **Gamma parametric distribution for OS** | | | | | |
| Lenvatinib | $64,134 | 1.13 | -$5,041 | 0.15 | Dominant |
| Sorafenib | $69,175 | 0.98 | - | - | - |
| **Log-logistic parametric distribution for PFS** | | | | | |
| Lenvatinib | $ 64,207 | 1.21 | -$4,921 | 0.18 | Dominant |
| Sorafenib | $ 69,129 | 1.03 | - | - | - |
| **Gamma parametric distribution for PFS** | | | | | |
| Lenvatinib | $ 64,239 | 1.20 | -$7,787 | 0.16 | Dominant |
| Sorafenib | $ 72,026 | 1.04 | - | - | - |

Utilities of 0.760 for progression-free and 0.680 for progression

Alternative utility values previously considered by pCODR in its reviews of regorafenib and nivolumab as second-line options for HCC were tested in a scenario analysis: 0.76 in the progression-free health state and 0.68 in the progressed health state [6, 26]. Incremental costs remain unchanged from the base-case, and there is a small increase in incremental QALYs. Lenvatinib remains dominant over sorafenib (Table 9).

Table S9: Alternative utility value scenario

| **Drug** | **Total Costs** | **Total QALYs** | **∆ Costs** | **∆ QALYs** | **ICUR** |
| --- | --- | --- | --- | --- | --- |
| Lenvatinib | $ 64,234 | 1.22 | -$5,021 | 0.18 | Dominant |
| Sorafenib | $ 69,255 | 1.04 | - | - | - |

Appendix 3: Univariate sensitivity analysis results†


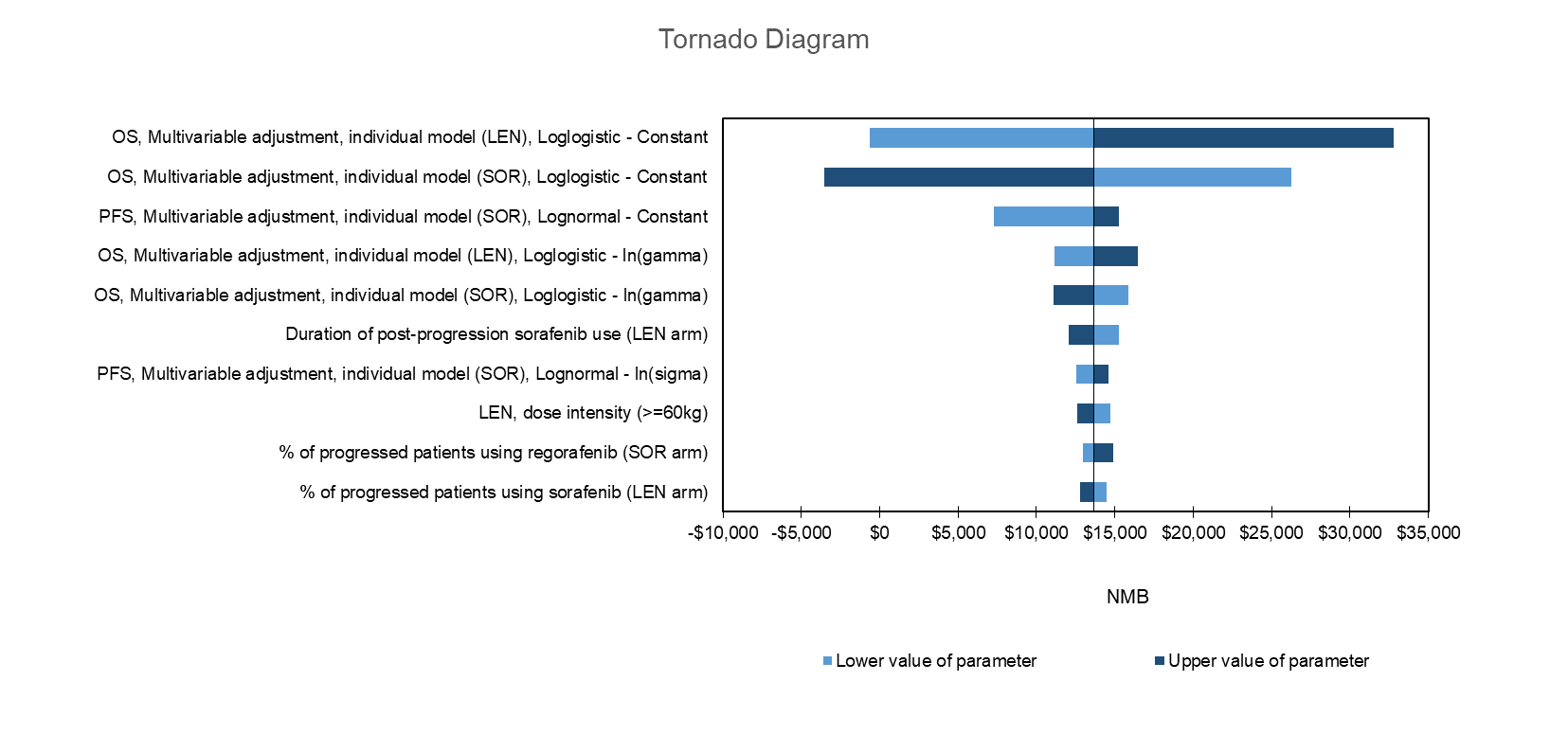


Abbreviations: LEN, lenvatinib; NMB, net monetary benefit; OS, overall survival; PFS, progression-free survival; SOR, sorafenib.
†Net monetary benefit is presented rather than the ICUR. Net monetary benefit is calculated as the incremental health benefit multiplied by the willingness-to-pay threshold, minus the incremental cost. This is to avoid situations with negative ICURs, which fail to distinguish between interventions that are less expensive and more effective or more expensive and less effective. A positive NMB always indicates that an intervention is cost-effective at a given willingness-to-pay threshold, and vice-versa, avoiding this ambiguity.
